# Consistency and Adequacy of Public and Commercial Health Insurance for US Children, 2016 to 2021

**DOI:** 10.1001/jamahealthforum.2023.4179

**Published:** 2023-11-22

**Authors:** Jamie R. Daw, Sarra Yekta, Faelan E. Jacobson-Davies, Stephen W. Patrick, Lindsay K. Admon

**Affiliations:** 1 Department of Health Policy and Management, Columbia University Mailman School of Public Health, New York, New York; 2 Department of Obstetrics and Gynecology, University of Michigan, Ann Arbor; 3 Departments of Pediatrics, Vanderbilt Center for Child Health Policy, Vanderbilt University Medical Center, Nashville, Tennessee; 4 Department of Health Policy, Vanderbilt Center for Child Health Policy, Vanderbilt University Medical Center, Nashville, Tennessee; 5 Institute for Healthcare Policy and Innovation, Department of Obstetrics and Gynecology, University of Michigan, Ann Arbor

## Abstract

**Importance:**

Before and during the COVID-19 public health emergency (PHE), commercially and publicly insured children may have faced different challenges in obtaining consistent and adequate health insurance.

**Objective:**

To compare overall rates, COVID-19 PHE-related changes, and child and family characteristics associated with inconsistent and inadequate coverage for publicly and commercially insured children.

**Design, Settings, and Participants:**

This was a cross-sectional study using nationally representative data from the 2016 to 2021 National Survey of Children’s Health of children from age 0 to 17 years living in noninstitutional settings.

**Exposure:**

Parent- or caregiver-reported current child health insurance type defined as public or commercial.

**Main Outcomes and Measures:**

Inconsistent insurance, defined as having an insurance gap in the past year; and inadequate insurance, defined by failure to meet 3 criteria: (1) benefits usually/always sufficient to meet child’s needs; (2) coverage usually/always allows child to access needed health care practitioners; and (3) no or usually/always reasonable annual out-of-pocket payments for child’s health care. Survey-weighted logistic regression was used to compare outcomes by insurance type, by year (2020-2021 vs 2016-2019), and by child characteristics within insurance type.

**Results:**

Of this nationally representative sample of 203 691 insured children, 34.5% were publicly insured (mean [SD] age, 8.4 [4.1] years; 47.4% female) and 65.5% were commercially insured (mean [SD] age, 8.7 [5.6]; 49.1% female). Most publicly insured children were either non-Hispanic Black (20.9%) or Hispanic (36.4%); living with 2 married parents (38.4%) or a single parent (33.1%); and had a household income less than 200% of the federal poverty level (79%). Most commercially insured children were non-Hispanic White (62.8%), living with 2 married parents (79.0%); and had a household income of 400% of the federal poverty level or higher (49.1%). Compared with commercially insured children, publicly insured children had higher rates of inconsistent coverage (4.2% vs 1.4%; difference, 2.7 percentage points [pp]; 95% CI, 2.3 to 3.2) and lower rates of inadequate coverage (12.2% vs 33.0%; difference, −20.8 pp; 95% CI, −21.6 to −20.0). Compared with the period from 2016 to 2019, inconsistent insurance decreased by 42% for publicly insured children and inadequate insurance decreased by 6% for commercially insured children during the COVID-19 PHE (2020-2021). The child and family characteristics associated with inadequate and inconsistent insurance varied by insurance type.

**Conclusions and Relevance:**

The findings of this cross-sectional study indicate that insurance gaps are a particular problem for publicly insured children, whereas insurance inadequacy and particularly, out-of-pocket costs are a challenge for commercially insured children. Both challenges improved during the COVID-19 PHE. Improving children’s health coverage after the PHE will require policy solutions that target the unique needs of commercially and publicly insured children.

## Key Points

**Question:**

How do the rates and child and family characteristics associated with inadequate and inconsistent health insurance coverage compare for publicly vs commercially insured children in the US?

**Findings:**

This cross-sectional study of 203 691 children found that publicly insured children experienced higher rates of inconsistent coverage, whereas commercially insured children faced higher rates of inadequate coverage. Public insurance consistency and commercial insurance adequacy improved substantially during the COVID-19 public health emergency.

**Meaning:**

The findings of this cross-sectional study suggest that policies are needed to address the unique issues faced by each population of insured children to improve the consistency and quality of children’s health coverage in the postpandemic context.

## Teaser

This cross-sectional study of commercially and publicly insured children in the US evaluates health coverage gaps, adequacy, and affordability comparing the periods before and during the COVID-19 public health emergency.

## Introduction

Consistent and adequate health insurance is critical to ensure children have access to affordable and high-quality health care. Children who have consistent coverage are more likely to attend well-child visits, have a usual source of care, and less likely to delay or forgo necessary medical care.^1,2,3^ Among insured children, having adequate insurance—coverage that offers affordable access to needed services and health care professionals—is associated with lower unmet health care needs and higher quality care.^4,5^ The health benefits of child insurance coverage have been shown to persist into adulthood, including lower rates of hospitalization, chronic conditions, and obesity.^6,7,8^

During the past 2 decades, the uninsured rate among children has steadily declined in the US. In 2021, 61.9% of US children were commercially insured, 36.4% were publicly insured, and only 5% were uninsured.^9^ This progress has been driven by policy reforms including expansions of Medicaid and the Children’s Health Insurance Program (CHIP); commercial insurance regulations on cost sharing and coverage for preventive services; the establishment of the Affordable Care Act (ACA) Health Insurance Marketplace and subsidies as well as other efforts to enhance outreach and streamline enrollment.^10^ However, alongside declines in uninsurance, the proportion of US children who had inconsistent or inadequate insurance increased from 30.6% in 2016 to 34.0% in 2019.^4^

Prior research has focused on documenting rates and trends in insurance consistency and adequacy at a national level for children covered by all insurance types^4,5,11^; however, the insurance-related challenges faced by publicly and commercially insured children are likely to differ due to differences in eligibility criteria, application processes, health care networks, cost-sharing requirements, as well as the accessibility, affordability, and quality of available care. The characteristics of children and families at greater risk of inconsistent or inadequate insurance may also vary by insurance type. Furthermore, policy responses during the COVID-19 public health emergency (PHE), such as continuous eligibility requirements for Medicaid and bolstered ACA Marketplace subsidies may have substantially affected children’s insurance. Although a national study found no change in US children’s insurance consistency and adequacy from 2019 to 2020 (the first year of the COVID-19 PHE),^11^ aggregate estimates may mask changes between publicly insured compared with commercially insured children, who were differentially affected by COVID-19 PHE policies.

Using nationally representative survey data from 2016 to 2021, the objective of this study was to evaluate the consistency and adequacy of health insurance for children insured by public compared with commercial insurance. We also compared changes during the COVID-19 PHE and identified sociodemographic and clinical characteristics associated with inconsistent and inadequate coverage within each insurance type.

## Methods

This study was deemed exempt by the institutional review board of the University of Michigan, and informed consent was waived because only deidentified data were used. We followed the Strengthening the Reporting of Observational Studies in Epidemiology (STROBE) reporting guideline.

### Study Sample

We conducted a secondary analysis of the 2016 to 2021 National Survey of Children’s Health (NSCH), a nationally representative survey of US children from birth to 17 years old living in noninstitutional settings. Our analysis used the NSCH topical survey, which includes more than 100 survey items focused on 1 child in the sampled household. Nearly all NSCH respondents (93.8%) were biological, foster, and adoptive parents or stepparents of the selected child; 5.8% were other relatives (eg, grandparents) and 0.3% were nonrelatives. Detailed information on the NSCH sampling and data collection procedures are available from the US Census Bureau.^12^

### Exposures

The primary exposure was child insurance type at the time of the NSCH survey. Public insurance included coverage from any form of government assistance including Medicaid and CHIP. Commercial insurance included coverage through a family member’s current or former employer or union, the ACA Marketplace, direct purchase from an insurance company, and TRICARE (the health care program of the US Department of Defense Military Health System) or other military health care. We excluded children who were covered by both public and commercial insurance (3.8% of the total sample) and those uninsured at the time of the survey (4.6%), including those insured only through the US Indian Health Service or through a religious health share.

### Outcomes

The 2 primary outcomes were (1) inconsistent insurance, defined as having an insurance gap in the past 12 months and (2) inadequate insurance, defined as coverage failing to meet the following criteria: (i) benefits were usually or always sufficient to meet child’s needs; (ii) coverage usually or always allowed child to see needed health care practitioners; and either (iii) no annual out-of-pocket (OOP) payments for child’s health care or (iv) OOP costs were usually or always reasonable. Secondary outcomes were the 4 individual criteria that comprised insurance adequacy. These outcome definitions are the National Performance Measures for the US Department of Health and Human Services Title V Maternal and Child Health Services Block Grant program and have been applied in prior research on health insurance using the NSCH.^4,11,13,14^

### Covariates

Sociodemographic characteristics of the child were reported by the adult NSCH respondent and included age, sex, race and/or ethnicity (non-Hispanic Black, Hispanic, non-Hispanic White, and other [including American Indian, Alaska Native, Asian Indian, Chinese, Filipino, Guamanian, Japanese, Korean, Native Hawaiian, Samoan, Vietnamese, other Pacific Islander, other Asian, some other race, and multiracial]), family structure (2 married parents; 2 unmarried parents, single parent, other), family income (as a percentage of the federal poverty level [FPL]), having at least 1 primary caregiver born in the US, and primary household language (English, Spanish, other). Clinical characteristics included whether the child had none, 1, or 2 or more of a list of 24 chronic physical or mental health conditions or disabilities, or any special health care needs (CSHCN).^12^ The NSCH CSHCN screener identifies children who require more than average use of health care services, counseling, or medications, or who experience a functional limitation due to a condition for a duration of 12 months or longer.^12^

### Statistical Analysis

We calculated rates of inconsistent and inadequate insurance overall and by sociodemographic and clinical characteristics stratified by insurance type. Because state Medicaid policies could drive heterogeneity in the outcomes, we also calculated rates of the outcomes for publicly insured children by state. To assess changes in the outcomes over time, we calculated rates by year and for the pooled prepandemic (2016-2019) and COVID-19 PHE (2020-2021) periods. We conducted unadjusted and adjusted logistic regressions to (1) compare outcome differences by insurance type, (2) estimate outcome changes during the PHE stratified by insurance type, and (3) identify child characteristics associated with the outcomes stratified by insurance type. For outcome differences by insurance type, we were primarily interested in unadjusted differences, which reflect both the unique populations served and the features of public compared with commercial insurance.

All analyses applied survey weights to produce nationally representative estimates that account for the NSCH sampling structure and nonresponse. The NSCH public-use files include imputed data for several variables.^12^ Child sex and race and/or ethnicity had low levels of missingness (<1%) and were imputed by the Census Bureau using hot-deck imputation. Household income had a high level of missingness (18%) and was inputted by the Census Bureau using sequential regression imputation. We used the Stata multiple imputation command to appropriately estimate means and variance based on the 6 imputed income values in the public-use data set. For all other variables with missing values not imputed by the Census Bureau, we included missing as a category in the analysis.

Statistical tests were 2-tailed and *P* < .05 was considered statistically significant. Data analyses were performed from March to August 2023.

## Results

The sample included 203 691 insured children (mean [SD] age, 8.6 [5.2] years; 98 511 [48.9%] female and 105 180 [51.1%] male) representing an annual average of 63.5 million children nationally during the study period from 2016 to 2021. Of these, 45 671 were publicly insured (weighted, 34.5%) and 158 020 were commercially insured (weighted, 65.5%) (Table 1). The child age distribution was similar across insurance types. Most publicly insured children were non-Hispanic Black (20.9%) or Hispanic (36.4%); living with 2 married parents (38.4%) or a single parent (33.1%); with a household income of less than 200% FPL (79.0%). Most commercially insured children were non-Hispanic White (62.8%), living with 2 married parents (79.0%), with a household income of 400% FPL or higher (49.1%). Compared with the commercially insured, more publicly insured children had multiple chronic conditions or disabilities (22.9% vs 15.3%) and any CSHCNs (23.9% vs 16.4%).

**Table 1.** Sample Characteristics by Child’s Current Health Insurance Type, 2016 to 2021

Characteristic	No. (weighted %)	
	Public (n = 45 671)	Commercial (n = 158 020)
Age, y		
<1	1764 (5.5)	5308 (4.7)
1-5	13 275 (28.3)	43 781 (27.2)
6-12	17 216 (40.3)	54 601 (39.1)
13-17	13 416 (25.9)	54 330 (28.9)
Sex		
Male	23 924 (51.6)	81 256 (50.9)
Female	21 747 (48.4)	76 764 (49.1)
Race and ethnicity		
Black, non-Hispanic	6025 (20.9)	6053 (8.3)
Hispanic	9550 (36.4)	14 257 (17.3)
White, non-Hispanic	23 793 (33.2)	117 520 (62.8)
Other^a^	6303 (9.5)	20 190 (11.6)
Family structure		
2 Parents, married	17 247 (38.4)	128 769 (79.0)
2 Parents, unmarried	5650 (13.6)	6301 (4.6)
1 Parent	15 939 (33.1)	18 639 (12.5)
Other caregiver	5507 (11.5)	1715 (1.6)
Missing data	1328 (3.4)	2596 (2.2)
Family income, % FPL		
<100	15 713 (42.4)	6122 (5.1)
100-199	16 862 (36.6)	13 940 (11.5)
200-299	7502 (12.5)	24 293 (17.7)
300-399	2808 (4.2)	27 700 (16.7)
≥400	2786 (4.2)	85 965 (49.1)
Any US-born caregiver		
Yes	37 225 (70.0)	142 563 (85.6)
No	5195 (20.1)	10 768 (10.3)
Missing data	3251 (9.9)	4689 (4.1)
Primary household language		
English	39 305 (74.7)	150 620 (92.7)
Spanish	3913 (18.6)	2179 (3.4)
Other	2070 (5.4)	4594 (3.5)
Missing data	383 (1.3)	627 (0.4)
Chronic conditions and disabilities, No.		
0	24 312 (59.3)	96 670 (64.5)
1	8508 (17.9)	33 743 (20.3)
≥2	12 851 (22.9)	27 607 (15.3)
CSHCN		
None	31 853 (76.1)	126 233 (83.6)
Any	13 818 (23.9)	31 787 (16.4)

Abbreviations: CSHCN, children with special health care needs; FPL, federal poverty level.

^a^ Other race or ethnicity included Alaska Native, American Indian, Asian Indian, Chinese, Filipino, Guamanian, Japanese, Korean, Native Hawaiian, Samoan, Vietnamese, other Pacific Islander, other Asian, any other race, and multiracial.

Unweighted sample sizes and survey-weighted prevalence estimates. Variables without a missing category had no missing values.

Table 2 shows inconsistent and inadequate insurance rates by insurance type. Publicly insured children were more likely than commercially insured children to have inconsistent coverage in the past year (4.2% vs 1.4%; unadjusted difference, 2.9 percentage points [pp]; 95% CI, 2.3 to 3.2 pp); however, they were less likely to have inadequate coverage (12.2% vs 33.0%; difference, −20.8 pp; 95% CI, −21.6 to −20.0 pp). Compared with commercially insured children, publicly insured children had higher rates of coverage that was always sufficient to meet the child’s needs (72.5% vs 64.1%; difference, 8.3 pp; 95% CI, 7.3 to 9.4]), no or low (<$250) annual OOP costs (91.5% vs 43.2%; difference, 48.3 pp; 95% CI, 47.5 to 49.0 pp), and always having reasonable OOP costs (84.3% vs 30.5%; difference, 53.8 pp; 95% CI, 52.9 to 54.6 pp) but had similar rates of always having coverage that allows the child to see needed health care practitioners (78.3% vs 77.3%; difference, 1.0 pp; 95% CI, 0.04 to 2.0). Adjusted differences were generally similar in magnitude and significance; however, after accounting for child characteristics, publicly insured children had higher rates of reporting coverage that allows the child to see needed health care practitioners (difference: 4.5 pp; 95% CI, 3.2 to 5.7 pp).

**Table 2.** Prevalence of Health Insurance Consistency and Adequacy Among US Children, by Insurance Type, 2016 to 2021

Outcome	% (95% CI)		Public vs commercial difference, percentage points (95% CI)	
	Public	Commercial	Unadjusted	Adjusted
Inconsistent coverage	4.2 (3.7 to 4.6)	1.4 (1.3 to 1.6)	2.9 (2.3 to 3.2)^a^	1.6 (1.0 to 2.1)^a^
Inadequate coverage	12.2 (11.6 to 12.9)	33.0 (32.5 to 33.5)	−20.8 (−21.6 to −20.0)^a^	−23.7 (−24.8 to −22.6)^a^
Inadequate/inconsistent coverage	15.0 (14.3 to 15.7)	33.6 (33.1 to 34.1)	−18.6 (−19.5 to −17.7)^a^	−21.8 (−23.0 to −20.6)^a^
**Coverage adequacy indicators**				
Sufficient to meet child’s needs				
Always	72.5 (71.5 to 73.4)	64.1 (63.6 to 64.7)	8.3 (7.3 to 9.4)^a^	11.4 (10.0 to 12.8)^a^
Usually	21.6 (20.8 to 22.5)	28.7 (28.3 to 29.2)	−7.1 (−8.1 to −6.2)^a^	−6.9 (−8.2 to −5.6)^a^
Never/sometimes	5.4 (4.9 to 5.9)	6.6 (6.3 to 6.9)	−1.2 (−1.8 to −0.7)^a^	−4.0 (−4.7 to −3.3)^a^
Allows child to visit needed health care practitioner				
Always	78.3 (77.5 to 79.2)	77.3 (76.9 to 77.8)	1.0 (0.04 to 2.0)^a^	4.5 (3.2 to 5.7)^a^
Usually	17.0 (16.3 to 17.8)	18.7 (18.2 to 19.1)	−1.6 (−2.5 to −0.7)	−3.3 (−4.5 to −2.1)^a^
Never/sometimes	3.9 (3.5 to 4.3)	3.5 (3.3 to 3.7)	0.4 (−0.01 to 0.9)	−1.1 (−1.7 to −0.6)^a^
Annual OOP costs, $				
0 or <250	91.5 (91.0 to 92.0)	43.2 (42.7 to 43.7)	48.3 (47.5 to 49.0)^a^	42.7 (41.5 to 43.9)^a^
250-499	3.7 (3.4 to 4.1)	20.3 (19.9 to 20.8)	−16.6 (−17.1 to −16.1)^a^	−14.4 (−15.2 to −13.6)^a^
500-999	2.0 (1.7 to 2.2)	15.0 (14.7 to 15.4)	−13.1 (−13.5 to −12.6)^a^	−11.5 (−12.1 to −10.8)^a^
1000-5000	1.5 (1.3 to 1.8)	17.3 (16.9 to 17.7)	−15.7 (−16.2 to −15.3)^a^	−14.1 (−14.7 to −13.5)^a^
>5000	0.3 (0.2 to 0.5)	3.2 (3.0 to 3.4)	−2.9 (−3.1 to −2.6)^a^	−2.6 (−2.9 to −2.3)^a^
Reasonable OOP costs				
Always	84.3 (83.6 to 85.0)	30.5 (30.0 to 31.0)	53.8 (52.9 to 54.6)^a^	48.7 (47.3 to 50.1)^a^
Usually	7.9 (7.4 to 8.4)	37.1 (36.6 to 37.6)	−29.1 (−29.9 to −28.4)^a^	−22.4 (−23.6 to −21.3)^a^
Never/sometimes	6.9 (6.4 to 7.4)	31.3 (30.8 to 31.7)	−24.3 (−25.0 to −23.6)^a^	−25.4 (−26.4 to −24.5)^a^

Abbreviation: OOP, out-of-pocket costs.

^a^ *P* < .05.

Survey-weighted prevalence estimates and differences. Missing percentages for all variables are less than 1% and not shown. Adjusted models include child age, sex, race and/or ethnicity, family structure, family income, caregiver born in the US, household language, chronic conditions and disabilities, and special health care needs.

### Changes During the COVID-19 PHE

The Figure shows inconsistent and inadequate coverage rates by year and insurance type. Among publicly insured children, inconsistent insurance decreased from 4.8% before the PHE to 2.9% during the PHE (adjusted difference, −2.0 pp; 95% CI, −2.8 to −1.2 pp; 42% decline from baseline). Among commercially insured children, inadequate insurance decreased from 33.6% to 31.7% during the PHE (adjusted difference, −2.0 pp; 95% CI, −2.9 to −1.0 pp; 5.9% decline from baseline), primarily due to improvements in reasonable OOP costs (eTable 2 in Supplement 1). No PHE-related changes were identified for public insurance adequacy or commercial insurance consistency.

**Figure.**
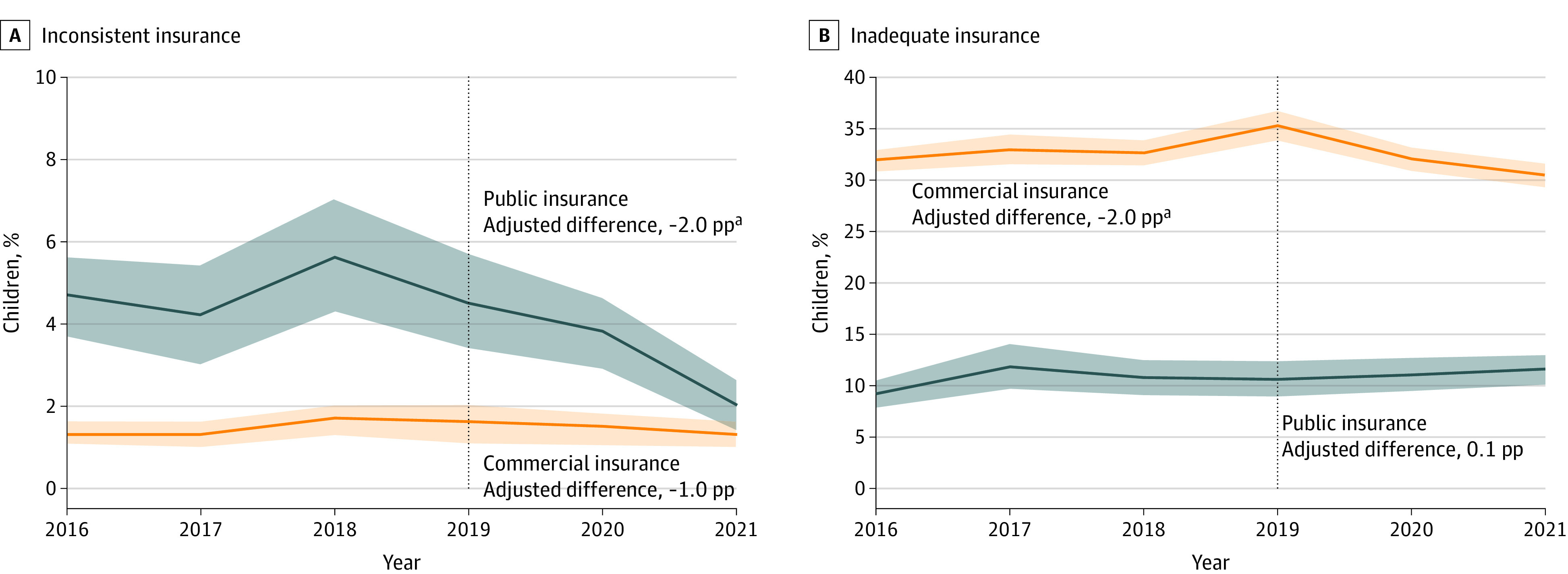
Inconsistent and Inadequate Health Insurance Coverage for US Children, by Year and Insurance Type, 2016 to 2021 ^a^*P* < .05. Survey-weighted prevalence estimates. Shaded areas represent 95% CIs . Adjusted models compare the outcomes during the COVID-19 public health emergency (2020-2021) to before the pandemic (2016-2019) adjusting for child age, sex, race and/or ethnicity, family structure, family income, US-born caregiver, household language, chronic conditions and disabilities, and special health care needs. Percentage points are indicated by pp.

### Child Characteristics Associated With Inconsistent and Inadequate Coverage

In adjusted models among publicly insured children, inconsistent coverage was significantly higher among Hispanic children and those with household incomes from 200% to 399% FPL (Table 3). Although differences by age category were not statistically significant, when comparing inconsistent insurance rates by year of age, we found that publicly insured children had markedly higher rates (7.3%) of inconsistent coverage at 1 year of age (eFigure 1 in Supplement 1). Inadequate coverage increased with child age and was significantly higher among publicly insured children with household incomes of 200% to 299% FPL and those with multiple chronic conditions and disabilities. Inconsistent coverage among publicly insured children varied from 0.6% in Vermont to 7.7% in Georgia, while inadequate coverage varied from 7.6% in Maine to 19.7% in Illinois (eTable 4, eFigures 2 and 3 in Supplement 1).

**Table 3.** Adjusted Predicted Probabilities of Inconsistent and Inadequate Health Coverage for Publicly Compared With Commercially Insured Children, 2016 to 2021

Characteristic	Adjusted predicted probability (95% CI)			
	Public		Commercial	
	Inconsistent coverage	Inadequate coverage	Inconsistent coverage	Inadequate coverage
Age, y				
<1	3.3 (1.2 to 5.4)^a^	6.6 (4.9 to 8.3)^a^	2.1 (1.1 to 3.0)^a^	40.0 (37.3 to 42.7)^a^
1-5	4.7 (3.7 to 5.7)	10.9 (9.6 to 12.1)^b^	1.7 (1.5 to 2.0)	31.1 (30.1 to 32.1)^b^
6-12	4.0 (3.4 to 4.7)	12.1 (11.1 to 13.1)^b^	1.4 (1.2 to 1.6)	33.0 (32.2 to 33.8)^b^
13-17	3.9 (3.3 to 4.6)	14.7 (13.3 to 16.1)^b^	1.2 (1 to 1.4)^b^	33.7 (32.8 to 34.5)^b^
Sex				
Male	4.1 (3.5 to 4.6)^a^	11.6 (10.8 to 12.5)^a^	1.4 (1.2 to 1.6)^a^	32.4 (31.7 to 33.1)^a^
Female	4.3 (3.6 to 4.9)	12.8 (11.8 to 13.8)	1.5 (1.3 to 1.7)	33.6 (32.9 to 34.3)^b^
Race and/or ethnicity				
Black, non-Hispanic	4.0 (3.2 to 4.9)	10.8 (9.4 to 12.1)	1.8 (1.2 to 2.4)^b^	30.1 (28.1 to 32.1)^b^
Hispanic	5.0 (3.8 to 6.2)^b^	13.0 (11.5 to 14.6)	1.6 (1.1 to 2.1)	35.5 (33.6 to 37.3)^b^
White, non-Hispanic	3.6 (3.1 to 4.2)^a^	12.2 (11.2 to 13.2)^a^	1.3 (1.1 to 1.4)^a^	33.2 (32.7 to 33.8)^a^
Other^c^	3.1 (2.3 to 4.0)	12.2 (10.3 to 14.0)	1.8 (1.3 to 2.3)^b^	30.4 (29.0 to 31.8)^b^
Family structure				
2 Parents, married	4.5 (3.7 to 5.3)^a^	12.4 (11.3 to 13.6)^a^	1.3 (1.2 to 1.5)^a^	32.8 (32.2 to 33.4)^a^
2 Parents, unmarried	3.7 (2.7 to 4.6)	10.8 (9.0 to 12.6)	2.2 (1.2 to 3.3)^b^	38.0 (35.1 to 40.8)^b^
1 Parent	4.6 (3.7 to 5.5)	13.6 (12.3 to 14.9)	1.8 (1.4 to 2.2)^b^	34.4 (32.9 to 35.8)^b^
Other	3.0 (1.9 to 4)^b^	8.9 (7.6 to 10.2)^b^	1.8 (0.8 to 2.8)	27.2 (22.3 to 32.2)^b^
Family income, % FPL				
<100	3.6 (2.9 to 4.2)^a^	11.2 (10.1 to 12.2)^a^	2.6 (1.7 to 3.6)^a^	38.0 (35.2 to 40.8)^a^
100-199	4.2 (3.4 to 5.0)	12.5 (11.3 to 13.7)	2.5 (2.0 to 3.0)	36.0 (34.2 to 37.8)
200-299	5.0 (3.7 to 6.2)^b^	14.5 (12.7 to 16.3)^b^	2.1 (1.7 to 2.5)	37.6 (36.2 to 39)
300-399	7.0 (3.4 to 10.6)^b^	12.2 (9.4 to 15)	1.6 (1.2 to 2.1)^b^	33.6 (32.4 to 34.8)^b^
≥400	4.9 (2.9 to 6.9)	13.7 (10.2 to 17.2)	0.7 (0.6 to 0.8)^b^	29.9 (29.3 to 30.6)^b^
Any US-born caregiver				
Yes	4.6 (3.1 to 6.0)^a^	13.7 (11.3 to 16.1)^a^	1.3 (0.9 to 1.8)^a^	38.4 (36.1 to 40.8)^a^
No	4.1 (3.4 to 4.7)	11.4 (10.5 to 12.4)	1.5 (1.3 to 1.6)	32.3 (31.7 to 32.9)
Household language				
English	4.3 (3.6 to 4.9)^a^	11.8 (10.9 to 12.7)^a^	1.4 (1.2 to 1.5)^a^	32.9 (32.4 to 33.5)^a^
Spanish	3.9 (2.5 to 5.4)	13.4 (10.7 to 16.1)	1.9 (1.0 to 2.8)	33.0 (28.6 to 37.4)
Other	3.9 (1.2 to 6.7)	13.8 (10.1 to 17.5)	2.4 (1.3 to 3.5)^b^	34.1 (30.9 to 37.2)
Chronic conditions and disabilities				
0	4.1 (3.4 to 4.7)^a^	10.7 (9.8 to 11.6)^a^	1.3 (1.1 to 1.5)^a^	30.2 (29.5 to 30.8)^a^
1	4.1 (3.3 to 5.0)	12.3 (10.7 to 14.0)	1.6 (1.2 to 1.9)	34.8 (33.7 to 35.9)^b^
≥2	4.3 (3.1 to 5.5)	15.6 (13.8 to 17.4)^b^	1.8 (1.4 to 2.3)^b^	42.2 (40.6 to 43.7)^b^
CSHCN				
None	4.5 (3.4 to 5.7)^a^	13.9 (12.2 to 15.6)^a^	1.5 (1.1 to 1.8)^a^	38.7 (37.3 to 40.1)^a^
Any	4.0 (3.5 to 4.6)	11.6 (10.8 to 12.4)^b^	1.4 (1.3 to 1.6)	31.8 (31.2 to 32.4)^b^

Abbreviations: CSHCN, children with special health care needs; FPL, federal poverty level.

^a^ Reference group.

^b^ Difference compared with reference group is statistically significant (*P* < .05).

^c^ Other race or ethnicity included Alaska Native, American Indian, Asian Indian, Chinese, Filipino, Guamanian, Japanese, Korean, Native Hawaiian, Samoan, Vietnamese, other Pacific Islander, other Asian, any other race, and multiracial.

Survey-weighted predicted probabilities and 95% CIs based on adjusted logistic regression models stratified by child health insurance type.

In adjusted models among commercially insured children, inconsistent coverage was significantly higher for non-Hispanic Black children, children of other race or ethnicity, as well as children in households with unmarried parents, single parents, lower incomes, and those with multiple chronic conditions and disabilities. In contrast to publicly insured children, differences in inadequate coverage were identified for most commercially insured child characteristics including age, sex, race and/or ethnicity, family structure, income, and health needs. Inadequate coverage was particularly high for commercially insured children younger than 1 year (adjusted predicted probability, 40.0%), children with household income less than 100% FPL (38.0%) and those with 2 or more chronic conditions and disabilities (42.2%). eTable 3 in Supplement 1 provides the unadjusted differences by child characteristics and insurance type.

## Discussion

Using nationally representative data, we found that inconsistent coverage is 3 times higher among publicly insured compared with commercially insured children. However, inadequate insurance is more prevalent overall, affecting nearly 1 in 5 children (16.5 million annually) in the US, with particularly high rates among the commercially insured. We also identified substantial improvements in public insurance consistency and commercial insurance adequacy during the COVID-19 PHE. Furthermore, we found that the child and family characteristics associated with higher rates of inconsistent and inadequate coverage differed by insurance type.

Consistent with prior research showing that insurance gaps are a particular issue for publicly insured children,^3,15,16^ we found that 4.2% of publicly insured children had a gap in the past year compared with only 1.4% of commercially insured children. Although some gaps are due to household income changes, a substantial share are for procedural reasons and nearly half of children who lose Medicaid-CHIP re-enroll within 12 months.^15^ As evidence of this, we found a notable spike in insurance gaps among publicly insured children at 1 year of age (7.3%), which reflects the first point of eligibility determination for most publicly insured children—given that being born to a mother with Medicaid automatically covers the child until their first birthday.

Compared with 2016 to 2019, we found that inconsistent public insurance declined by 42% during the PHE when continuous Medicaid eligibility requirements were in place. The unwinding of these protections in 2023 is projected to leave 5.3 million children without Medicaid-CHIP coverage, potentially resulting in delays and forgone care.^17^ Among disenrolled children, 74% are projected to be disenrolled despite being eligible for Medicaid.^17^ The remaining 26% of Medicaid ineligible children will need to enroll in commercial coverage, which our findings indicate offers less adequate coverage, particularly for low- and middle-income families.

States have promising policy options to bridge insurance gaps for publicly insured children in the post-PHE context. Since 1997, the US Centers for Medicare & Medicaid Services (CMS) has allowed states to grant 12-month continuous Medicaid-CHIP eligibility for children, which has been associated with reduced insurance gaps.^18^ However, as of January 2023, only 23 states have implemented this policy.^19^ In 2022, CMS approved Oregon’s 1115 waiver that allows children to continuously maintain Medicaid-CHIP from birth until age 6 years, with 2-year continuous eligibility from age 6 to 17 years. Although multiyear continuous eligibility rules are the most durable approaches to improve coverage consistency, states can also address procedural disenrollment through automatic renewal, increased funding for consumer assistance, and working with managed care plans to maintain updated beneficiary contact information.^20^ Recent proposed rulemaking from CMS would make it easier for states to implement these streamlined enrollment and renewal intitiatives.^21^

Our findings also point to a particular need for state Medicaid programs to conduct targeted outreach and linguistically and culturally competent navigation assistance for immigrant families. We found that 1 in 20 publicly insured Hispanic children had inconsistent coverage in the past year, with similar rates among children without a US-born caregiver (4.6%). These findings could partly reflect reticence among immigrant parents to enroll eligible children in Medicaid-CHIP for fear of immigration-related consequences. Although the Biden Administration reversed the Trump Administration’s public charge inadmissibility rule for Medicaid (effective from 2019-2022), the documented effects of this policy on child insurance enrollment are likely to persist and additional efforts will be required to redevelop trust.^22^

Among commercially insured children, inconsistent coverage was low overall (1.4%); however, the highest rates (approximately 2.5%) were among low-income households (<300% FPL) who are also eligible for Medicaid-CHIP in most states. Thus, the policy solutions to reduce inconsistent commercial coverage may also lie with Medicaid-CHIP reforms. For example, allowing CHIP to act as a secondary payer for income-eligible families regardless of other coverage could reduce insurance churn, particularly for low-income working families who may move between jobs with and without coverage offerings.

Despite the important opportunities to reduce insurance gaps among US children, our findings suggest that there is a need for renewed attention and investment in policies to improve insurance adequacy. We found that inadequate coverage affects 1 in 8 publicly insured children (2.7 million annually) and 1 in 3 commercially insured children (13.8 million annually), a far larger share than that affected by inconsistent coverage. Children with medically complex conditions who were covered by commercial insurance plans were the most vulnerable—42% of children with multiple chronic conditions reported inadequate coverage.

The primary driver of inadequate insurance for commercially insured children is affordability: only 30% reported always having reasonable OOP costs and 20% reported annual spending of $1000 or more. Public insurance provides comparably strong financial protection, with 84% reporting reasonable OOP costs and 92% reporting no costs or costs under $250. There are 3 primary avenues to improve affordability of care for commercially insured children. One direction involves expanding Medicaid-CHIP to shift more children to public coverage— by setting higher federal standards for state Medicaid income eligibility for children (eg, 300% or 400% FPL vs currently 138%) and/or allowing families above the Medicaid-CHIP cutoff to buy into the program with corresponding maximum limits on premiums and cost sharing to ensure affordability.^23^ Whereas adult Medicaid beneficiaries typically report higher barriers to accessing care compared with commercially insured adults,^24^ our results showed that commercially insured and publicly insured children were equally or more likely to report always having coverage to see needed health care practitioners. Thus, it is possible that moving more children to public insurance may not affect access, although a large shift may require increased Medicaid reimbursement to ensure adequate participation by health care practitioners and facilities.

Other strategies to improve insurance adequacy include bolstering subsidies for ACA Marketplaces (overall or specifically for families with children), broadening benefit requirements for health plans, particularly for children with CSHCN, enhancing pediatric network requirements, and allowing Marketplace enrollees to hold supplementary Medicaid coverage.^20,23^ Indeed, we found that inadequate commercial coverage declined by 6% during the PHE alongside reduced OOP costs, which could reflect the availability of more generous Marketplace subsidies during the PHE (recently extended through 2025).

### Limitations

Study limitations included outcomes based on caregiver reports, which could be subject to differential recall or reporting bias across population subgroups or insurance type. Second, insurance adequacy could be conceptualized more broadly; for example, the NSCH measure did not include quality of care or the administrative burden of maintaining and using insurance. Third, we excluded children with both commercial and public insurance owing to small sample size; however, this may have disproportionally excluded children with special needs who are more likely to be dually insured. Lastly, the NSCH data did not include commercial plan details (eg, deductibles, cost sharing levels), which likely masked heterogeneity in commercial insurance adequacy.

## Conclusions

This cross-sectional study found that publicly insured children have higher rates of inconsistent coverage but considerably lower rates of inadequate coverage compared with commercially insured children. Inadequate coverage affects a far large share of US children overall, primarily due to high OOP costs for those with commercial insurance. Policies are urgently needed to maintain and build on PHE-era gains in public insurance consistency and commercial insurance adequacy to ensure that all US children have affordable access to high-quality health care.

## Notes

Supplement 1. **eTable 1.** Insurance Consistency and Adequacy by Year and Pre (2016-19) vs. Post-Pandemic (2020-21) Differences for Publicly Insured Children **eTable 2.** Insurance Consistency and Adequacy by Year and Pre (2016-19) vs. Post-Pandemic (2020-21) Differences for Commercially Insured Children **eFigure 1.** Inconsistent and Inadequate Insurance by Child Age and Insurance Type, US 2016-2021 **eTable 3.** Unadjusted Predicted Probabilities of Inconsistent and Inadequate Coverage for Publicly and Commercially Insured Children, US 2016-2021. **eTable 4.** Inconsistent and Inadequate Insurance for Publicly Insured Children by State, 2016-2021 **eFigure 2.** Inconsistent Insurance for Publicly Insured Children by State, 2016-2021 **eFigure 3.** Inadequate Insurance for Publicly Insured Children by State, 2016-2021

Supplement 2.
Data Sharing Statement

## References

[1] Cassedy A, Fairbrother G, Newacheck PW. The impact of insurance instability on children’s access, utilization, and satisfaction with health care. Ambul Pediatr. 2008;8(5):321-328. doi:10.1016/j.ambp.2008.04.007 18922506

[2] Federico SG, Steiner JF, Beaty B, Crane L, Kempe A. Disruptions in insurance coverage: patterns and relationship to health care access, unmet need, and utilization before enrollment in the State Children’s Health Insurance Program. Pediatrics. 2007;120(4):e1009-e1016. doi:10.1542/peds.2006-3094 17908722

[3] Buchmueller T, Orzol SM, Shore-Sheppard L. Stability of children’s insurance coverage and implications for access to care: evidence from the Survey of Income and Program Participation. Int J Health Care Finance Econ. 2014;14(2):109-126. doi:10.1007/s10754-014-9141-1 24504692

[4] Yu J, Perrin JM, Hagerman T, Houtrow AJ. Underinsurance among children in the United States. Pediatrics. 2022;149(1):e2021050353. doi:10.1542/peds.2021-050353 34866156 PMC9647940

[5] Kogan MD, Newacheck PW, Blumberg SJ, . Underinsurance among children in the United States. N Engl J Med. 2010;363(9):841-851. doi:10.1056/NEJMsa0909994 20818845

[6] Institute of Medicine (US) Committee on Health Insurance Status and Its Consequences. America’s Uninsured Crisis: Consequences for Health and Health Care. National Academies Press; 2009. Accessed May 16, 2023.https://www.ncbi.nlm.nih.gov/books/NBK214966/ 25009923

[7] Boudreaux MH, Golberstein E, McAlpine DD. The long-term impacts of Medicaid exposure in early childhood: Evidence from the program’s origin. J Health Econ. 2016;45:161-175. doi:10.1016/j.jhealeco.2015.11.001 26763123 PMC4785872

[8] Miller S, Wherry LR. The long-term effects of early life Medicaid coverage. J Hum Resour. 2018. doi:10.3368/jhr.54.3.0816.8173R1

[9] US Census Bureau UC. More Children Were Covered by Medicaid and CHIP in 2021. Published 2022. Accessed May 16, 2023. https://www.census.gov/library/stories/2022/09/uninsured-rate-of-children-declines.html

[10] Artiga S, Ubri P. Key Issues in Children’s Health Coverage. Kaiser Family Foundation; 2017. Accessed May 16, 2023. https://www.kff.org/medicaid/issue-brief/key-issues-in-childrens-health-coverage/

[11] Lebrun-Harris LA, Ghandour RM, Kogan MD, Warren MD. Five-year trends in US Children’s Health and Well-being, 2016-2020. JAMA Pediatr. 2022;176(7):e220056. doi:10.1001/jamapediatrics.2022.0056 35285883 PMC8922203

[12] US Census Bureau. NSCH Survey Methodology. Published 2022. Accessed May 16, 2023. https://www.childhealthdata.org/learn-about-the-nsch/methods

[13] Child and Adolescent Health Measurement Initiative. Title V Maternal and Child Health Services Block Grant Measures Content Map, 2016-2020 National Survey of Children’s Health (Five Years Combined), 2022. Accessed June 8, 2023. https://nschdata.org/App_Themes/Main/Contents/nsch/content-map/2016-2020_NSCH_Content_Map_NPMs_NOMs_CAHMI.pdf.

[14] Gaffney A, Dickman S, Cai C, McCormick D, Himmelstein DU, Woolhandler S. Medical uninsurance and underinsurance among US children: findings from the National Survey of Children’s Health, 2016-2019. JAMA Pediatr. 2021;175(12):1279-1281. doi:10.1001/jamapediatrics.2021.2822 34424273 PMC8383158

[15] Medicaid and CHIP Payment and Access Commission. An Updated Look at Rates of Churn and Continuous Coverage in Medicaid and CHIP; 2021. Accessed May 22, 2023. https://www.macpac.gov/publication/an-updated-look-at-rates-of-churn-and-continuous-coverage-in-medicaid-and-chip-abstract/

[16] Orzol SM, Hula L, Harrington M. Program churning and transfers between Medicaid and CHIP. Acad Pediatr. 2015;15(3)(suppl):S56-S63. doi:10.1016/j.acap.2015.02.006 25906961

[17] Office of Health Policy. Assistant Secretary for Planning and Evaluation. Unwinding the Medicaid Continuous Enrollment Provision: Projected Enrollment Effects and Policy Approaches. Published 2022. Accessed October 25, 2023. https://aspe.hhs.gov/sites/default/files/documents/dc73e82abf7fc26b6a8e5cc52ae42d48/aspe-end-mcaid-continuous-coverage.pdf

[18] Brantley E, Ku L. Continuous eligibility for Medicaid associated with improved child health outcomes. Med Care Res Rev. 2022;79(3):404-413. doi:10.1177/10775587211021172 34525877

[19] Kaiser Family Foundation. State Adoption of 12-Month Continuous Eligibility for Children's Medicaid and CHIP. Published 2023. Accessed May 22, 2023. https://www.kff.org/health-reform/state-indicator/state-adoption-of-12-month-continuous-eligibility-for-childrens-medicaid-and-chip

[20] Alker J, Brooks T. Millions of Children May Lose Medicaid: What Can Be Done to Help Prevent Them From Becoming Uninsured? Center for Children and Families. 2022. Accessed May 22, 2023. https://ccf.georgetown.edu/2022/02/17/millions-of-children-may-lose-medicaid-what-can-be-done-to-help-prevent-them-from-becoming-uninsured/

[21] US Centers for Medicare & Medicaid Services. Streamlining Eligibility & Enrollment: Notice of Proposed Rulemaking. Published 2022. Accessed May 22, 2023. https://www.cms.gov/newsroom/fact-sheets/streamlining-eligibility-enrollment-notice-propose-rulemaking-nprm

[22] Artiga S, Damico A, Garfield R. Potential Effects of Public Charge Changes on Health Coverage for Citizen Children. Kaiser Family Foundation; 2018. Accessed May 22, 2023. https://www.kff.org/racial-equity-and-health-policy/issue-brief/potential-effects-of-public-charge-changes-on-health-coverage-for-citizen-children/

[23] Alker JC, Kenney GM, Rosenbaum S. Children’s health insurance coverage: progress, problems, and priorities for 2021 and beyond. Health Aff (Millwood). 2020;39(10):1743-1751. doi:10.1377/hlthaff.2020.00785 33017236

[24] Hsiang WR, Lukasiewicz A, Gentry M, . Medicaid patients have greater difficulty scheduling health care appointments compared with private insurance patients: a meta-analysis. Inquiry. 2019;56:46958019838118. doi:10.1177/0046958019838118 30947608 PMC6452575

