# Supplementary material for: Consistency and Adequacy of Public and Commercial Health Insurance for US Children, 2016 to 2021
Source: JAMA Health Forum. 2023 Nov 22;4(11):e234179. doi: 10.1001/jamahealthforum.2023.4179 (PMC10665966; doi:10.1001/jamahealthforum.2023.4179)
Supplement: Supplement 1. — eTable 1. Insurance Consistency and Adequacy by Year and Pre (2016-19) vs. Post-Pandemic (2020-21) Differences for Publicly Insured Children eTable 2. Insurance Consistency and Adequacy by Year and Pre (2016-19) vs. Post-Pandemic (2020-21) Differences for Commercially Insured Children eFigure 1. Inconsistent and Inadequate Insurance by Child Age and Insurance Type, US 2016-2021 eTable 3. Unadjusted Predicted Probabilities of Inconsistent and Inadequate Coverage for Publicly and Commercially Insured Children, US 2016-2021. eTable 4. Inconsistent and Inadequate Insurance for Publicly Insured Children by State, 2016-2021 eFigure 2. Inconsistent Insurance for Publicly Insured Children by State, 2016-2021 eFigure 3. Inadequate Insurance for Publicly Insured Children by State, 2016-2021 [file jamahealthforum-e234179-s001.pdf]

Supplemental Online Content

Daw JR, Yekta S, Jacobson-Davies FE, Patrick SW, Admon LK. Consistency and Adequacy of Public and Commercial Health Insurance for US Children, 2016 to 2021. *JAMA Health Forum*. 2023;4(11):234179. doi:10.1001/jamahealthforum.2023.4179

eTable 1. Insurance Consistency and Adequacy by Year and Pre (2016-19) vs. Post-Pandemic (2020-21) Differences for Publicly Insured Children

eTable 2. Insurance Consistency and Adequacy by Year and Pre (2016-19) vs. Post-Pandemic (2020-21) Differences for Commercially Insured Children

eFigure 1. Inconsistent and Inadequate Insurance by Child Age and Insurance Type, US 2016-2021

eTable 3. Unadjusted Predicted Probabilities of Inconsistent and Inadequate Coverage for Publicly and Commercially Insured Children, US 2016-2021.

eTable 4. Inconsistent and Inadequate Insurance for Publicly Insured Children by State, 2016-2021

eFigure 2. Inconsistent Insurance for Publicly Insured Children by State, 2016-2021

eFigure 3. Inadequate Insurance for Publicly Insured Children by State, 2016-2021

This supplemental material has been provided by the authors to give readers additional information about their work.

© 2023 Daw JR et al. *JAMA Health Forum.*

eTable 1. Insurance Consistency and Adequacy by Year and Pre (2016-19) vs. Post-Pandemic (2020-21) Differences for Publicly-Insured Children

Outcomes	2016	2017	2018	2019	2016-2019 Pooled	2020	2021	2020-2021 Pooled	Difference (2020-21 vs. 2016-19)	
									**Unadjusted**	**Adjusted**
Inconsistent Coverage	4.7	4.2	5.6	4.5	4.8 (4.2, 5.4)	3.8	2.0	2.9 (2.3, 3.4)	-1.9 (-2.7, -1.1)*	-2.0 (-2.8, -1.2)*
Inadequate Coverage	10.7	13.2	12.2	12.0	12.0 (11.2, 12.8)	12.4	12.9	12.6 (11.6, 13.7)	0.6 (-0.7, 2.0)	0.1 (-1.2, 1.5)
Inadequate or Inconsistent Coverage	13.8	16.1	16.1	15.2	15.3 (14.3, 16.2)	14.9	13.9	14.4 (13.3, 15.5)	-0.9 (-2.4, 0.6)	-1.4 (-2.9, 0.01)
**Adequacy Indicators**										
Sufficient to meet child's needs										
Always	71.6	73.8	74.4	72.5	73.1 (71.9, 74.2)	71.5	70.9	71.2 (69.8, 72.6)	-1.9 (-3.7, -0.01)*	-1.4 (-3.2, 0.4)
Usually	22.9	19.9	19.9	21.8	21.1 (20.1, 22.2)	22.4	22.7	22.6 (21.3, 23.9)	1.4 (-0.3, 3.1)	1.1 (-0.5, 2.8)
Never/Sometimes	4.7	6.0	5.2	5.4	5.3 (4.7, 5.9)	5.6	5.6	5.6 (4.8, 6.4)	0.3 (-0.7, 1.3)	0.1 (-0.9, 1.2)
Allows child to see needed providers										
Always	79.5	79.7	80.2	77.9	79.3 (78.3, 80.4)	76.4	75.9	76.2 (74.8, 77.5)	-3.2 (-4.9, -1.4)*	-2.8 (-4.5, -1.0)*
Usually	15.9	15.1	16.1	17.8	16.2 (15.2, 17.2)	18.6	18.9	18.8 (17.5, 20.0)	2.6 (1.0, 4.2)*	2.3 (0.7, 3.8)*
Never/Sometimes	3.3	4.5	3.3	3.9	3.8 (3.3, 4.3)	4.4	4.0	4.2 (3.5, 4.9)	0.4 (-0.4, 1.3)	0.3 (-0.5, 1.2)
Annual OOP costs										
None or <$250	92.2	91.8	92.3	90.2	91.7 (91.0, 92.3)	91.0	91.2	91.1 (90.3, 91.9)	-0.6 (-1.6, 0.5)	0.9 (-1.1, 1.0)
$250-499	3.5	3.5	2.9	4.3	3.5 (3.1, 4.0)	3.8	4.5	4.1 (3.5, 4.8)	0.6 (-0.1, 1.3)	0.4 (-0.3, 1.1)
$500-999	1.5	1.9	1.6	2.7	1.9 (1.6, 2.3)	2.2	2.0	2.1 (1.7, 2.5)	0.2 (-0.3, 0.7)	0.0 (-0.5, 0.5)
$1000-5000	1.2	1.6	1.9	1.4	1.5 (1.2, 1.8)	1.8	1.4	1.6 (1.2, 2.0)	0.1 (-0.4, 0.6)	-0.1 (-0.6, 0.4)
>$5000	0.2	0.3	0.3	0.5	0.3 (0.1, 0.5)	0.3	0.3	0.3 (0.1, 0.4)	0.0 (-0.3, 0.2)	-0.1 (-0.3, 0.2)
Reasonable OOP costs										
Always	84.8	85.5	84.4	83.2	84.5 (83.6, 85.4)	83.5	84.2	83.9 (82.8, 84.9)	-0.6 (-2.0, 0.8)	0.3 (-1.1, 1.6)
Usually	7.8	6.8	7.4	8.8	7.7 (7.1, 8.3)	8.9	7.9	8.4 (7.6, 9.2)	0.7 (-0.4, 1.7)	0.3 (-0.8, 1.3)
Never/Sometimes	6.2	7.1	7.5	7.1	7.0 (6.3, 7.6)	6.6	7.0	6.8 (6.1, 7.5)	-0.1 (-1.1, 0.8)	-0.6 (-1.5, 0.4)

Notes: Survey-weighted prevalence estimates and differences. Adjusted models include child age, sex, race-ethnicity, family structure, family income, caregiver born in US, household language, chronic conditions and disabilities, and special health care needs. *p<0.05

© 2023 Daw JR et al. *JAMA Health Forum.*

eTable 2. Insurance Consistency and Adequacy by Year and Pre (2016-19) vs. Post-Pandemic (2020-21) Differences for Commercially-Insured Children

Outcomes	2016	2017	2018	2019	2016-2019 Pooled	2020	2021	2020-2021 Pooled	Difference (2020-21 vs. 2016-19)	
									**Unadjusted**	**Adjusted**
Inconsistent Coverage	1.3	1.3	1.7	1.6	1.5 (1.3, 1.6)	1.5	1.3	1.4 (1.2, 1.6)	-0.1 (-0.4, 0.2)	-0.1 (-0.3, 0.2)
Inadequate Coverage	32.4	33.4	33.1	35.6	33.6 (33.0, 34.3)	32.5	31.0	31.7 (31.0, 32.5)	-1.9 (-2.9, -0.9)*	-2.0 (-2.9, -1.0)*
Inadequate or Inconsistent Coverage	32.9	34.1	33.7	36.2	34.2 (33.6, 34.9)	33.1	31.5	32.3 (31.5, 33.1)	-1.9 (-2.9, -0.9)*	-2.0 (-3.0, -1.0)*
**Adequacy Indicators**										
Sufficient to meet child's needs										
Always	65.4	64.0	63.4	62.8	63.9 (63.2, 64.5)	65.4	64.0	64.7 (63.9, 65.5)	0.8 (-0.3, 1.8)	0.7 (-0.3, 1.7)
Usually	27.6	29.1	29.3	29.5	28.9 (28.3, 29.5)	27.9	29.0	28.4 (27.7, 29.2)	-0.5 (-1.4, 0.5)	-0.5 (-1.4, 0.5)
Never/Sometimes	6.4	6.4	6.9	7.1	6.7 (6.4, 7.1)	6.4	6.4	6.4 (6.0, 6.8)	-0.3 (-0.9, 0.2)	-0.3 (-0.8, 0.3)
Allows child to see needed providers										
Always	79.1	78.0	77.5	76.2	77.7 (77.1, 78.2)	77.7	75.5	76.6 (75.9, 77.3)	-1.1 (-2.0, -0.1)*	-1.1 (-2.0, -0.2)*
Usually	16.7	18.2	18.5	19.7	18.3 (17.8, 18.8)	18.5	20.2	19.4 (18.7, 20.0)	1.1 (0.2, 1.9)*	1.1 (0.2, 1.9)*
Never/Sometimes	3.4	3.5	3.4	3.7	3.5 (3.2, 3.7)	3.4	3.5	3.4 (3.1, 3.8)	-0.1 (-0.5, 0.4)	0.0 (-0.5, 0.4)
Annual OOP costs										
None or <$250	43.4	43.1	42.3	42.3	42.8 (42.1, 43.5)	44.3	43.8	44.1 (43.2, 44.9)	1.3 (0.2, 2.4)*	1.5 (0.5, 2.6)*
$250-499	21.1	19.8	20.2	21.1	20.6 (20.0, 21.1)	19.9	19.8	19.8 (19.2, 20.5)	-0.7 (-1.6, 0.1)	-0.7 (-1.6, 0.1)
$500-999	14.7	15.4	16.0	14.8	15.2 (14.8, 15.7)	14.3	14.9	14.6 (14.0, 15.2)	-0.7 (-1.4, 0.1)	-0.7 (-1.4, 0.1)
$1000-5000	16.7	17.6	17.7	17.4	17.3 (16.9, 17.8)	17.2	17.2	17.2 (16.6, 17.8)	-0.1 (-0.9, 0.6)	-0.3 (-1.1, 0.4)
>$5000	2.7	3.1	2.9	3.4	3.0 (2.8, 3.2)	3.5	3.4	3.5 (3.2, 3.8)	0.5 (0.1, 0.8)*	0.4 (0.0, 0.7)*
Reasonable OOP costs										
Always	29.6	30(2	28.6	29.6	29.4 (28.8, 30.1)	32.2	33.1	32.6 (31.8, 33.4)	3.2 (2.1, 4.2)*	3.3 (2.3, 4.3)*
Usually	38.4	37.1	39.1	35.5	37.5 (36.9, 38.2)	35.8	36.5	36.1 (35.4, 36.9)	-1.4 (-2.4, -0.4)*	-1.4 (-2.4, -0.4)*
Never/Sometimes	30.8	31.8	31.1	33.8	31.9 (31.3, 32.5)	30.9	29.1	30.0 (29.2, 30.7)	-1.9 (-2.9, -0.9)*	-2.0 (-3.0, -1.0)*

Notes: Survey-weighted prevalence estimates and differences. Adjusted models include child age, sex, race-ethnicity, family structure, family income, caregiver born in US, household language, chronic conditions and disabilities, and special health care needs. *p<0.05

© 2023 Daw JR et al. *JAMA Health Forum.*

eFigure 1. Inconsistent and Inadequate Insurance by Child Age and Insurance Type, US 2016-2021

(A) Inconsistent Insurance

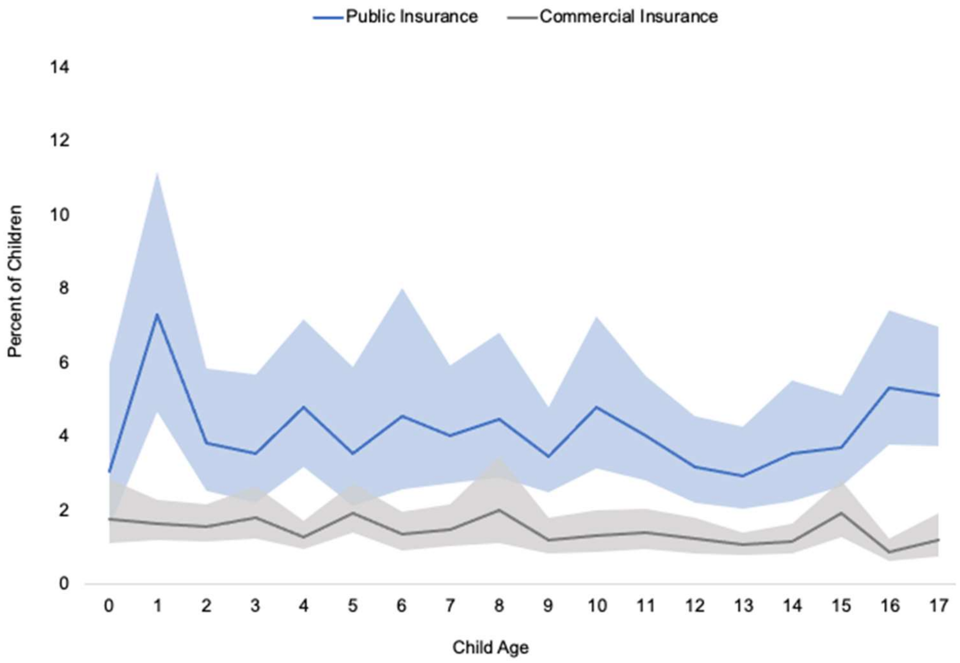

(B) Inadequate Insurance

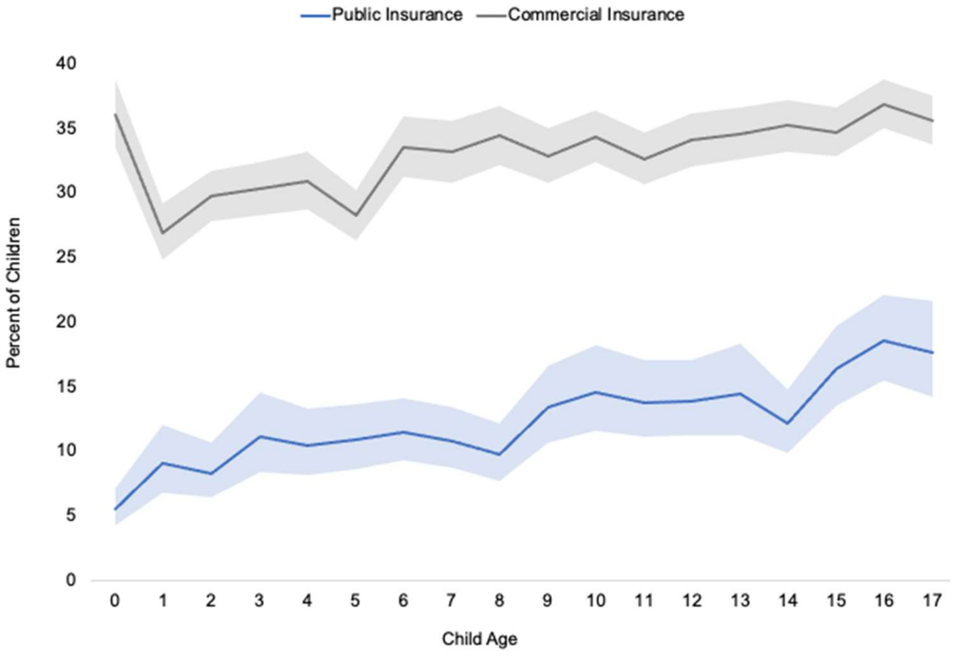

Notes: Survey-weighted prevalence estimates. Shaded areas represent 95% confidence intervals.

© 2023 Daw JR et al. *JAMA Health Forum.*

eTable 3. Unadjusted Predicted Probabilities of Inconsistent and Inadequate Coverage for Publicly- and Commercially-Insured Children, US 2016-2021.

	**Public** Unadjusted Predicted Probability (95% CI)		**Commercial** Unadjusted Predicted Probability (95% CI)	
**Characteristics**	**Inconsistent Coverage**	**Inadequate Coverage**	**Inconsistent Coverage**	**Inadequate Coverage**
**Child Age**				
<1	3.1 (1.6, 6.0)†	5.5 (4.2, 7.1)†	1.8 (1.1, 2.8)†	36.1 (33.5, 38.8)†
1-5	4.6 (3.7, 5.6)	10.0 (8.9, 11.2)*	1.6 (1.4, 1.9)	29.2 (28.3, 30.2)*
6-12	4.1 (3.5, 4.8)	12.5 (11.5, 13.6)*	1.4 (1.2, 1.7)	33.6 (32.8, 34.4)
13-17	4.1 (3.5, 4.8)	15.7 (14.3, 17.2)*	1.2 (1.0, 1.5)	35.4 (34.5, 36.3)
**Child Sex**				
Male	4.1 (3.5, 4.7)†	11.8 (11.0, 12.7)†	1.4 (1.2, 1.6)†	32.9 (32.2, 33.6)†
Female	4.3 (3.6, 5.0)	12.7 (11.7, 13.7)	1.5 (1.3, 1.7)	33.2 (32.5, 33.9)
**Child Race-Ethnicity**				
Black, Non-Hispanic	3.9 (3.2, 4.8)	10.4 (9.3, 11.7)	2.2 (1.6, 3.0)*	32.2 (30.2, 34.3)
Hispanic	4.9 (4.0, 6.0)*	13.8 (12.4, 15.3)*	2.0 (1.5, 2.6)*	36.9 (35.1, 38.8)*
White, Non-Hispanic	3.8 (3.4, 4.3)†	11.7 (10.9, 12.5)†	1.1 (1.0, 1.3)†	32.5 (32.0, 33.0)†
Other**	3.1 (2.4, 4.0)	12.1 (10.6, 13.8)	1.8 (1.4, 2.3)*	31.0 (29.6, 32.3)*
**Family Structure**				
Two parents, married	4.8 (4.0, 5.7)†	12.6 (11.4, 13.8)†	1.2 (1.1, 1.3)†	32.2 (31.6, 32.7)†
Two parents, unmarried	3.7 (2.7, 5.1)	10.1 (8.3, 12.3)*	2.8 (1.5, 5.2)*	38.9 (35.3, 42.6)*
Single parent	4.4 (3.7, 5.2)	13.1 (12.0, 14.3)	2.4 (1.9, 3.0)*	36.4 (34.9, 38.0)*
Other	3.5 (2.8, 4.3)	10.6 (9.4, 11.9)*	1.9 (1.5, 2.4)*	34.6 (32.8, 36.5)*
**Family Income**				
<100% FPL	3.6 (3.0, 4.3)†	11.3 (10.2, 12.4)†	3.1 (2.0, 4.1)†	38.4 (35.5, 41.2)†
100-199% FPL	4.2 (3.4, 5.0)	12.6 (11.4, 13.9)	2.7 (2.1, 3.3)	36.4 (34.6, 38.2)
200-299% FPL	4.8 (3.6, 6.0)	14.2 (12.4, 15.9)*	2.2 (1.8, 2.6)	37.9 (36.4, 39.3)
300-399% FPL	6.6 (3.1, 10.1)	11.1 (8.5, 13.8)	1.6 (1.1, 2.0)	33.6 (32.3, 34.8)*
>=400% FPL	4.4 (2.7, 6.1)	12.7 (9.4, 16.1)	0.7 (0.6, 0.8)	29.7 (29.1, 30.4)*
**Any Caregiver Born in US**				
Yes	4.1 (3.7, 4.6)†	11.2 (10.5, 11.9)†	1.4 (1.2, 1.5)†	32.6 (32.1, 33.1)†
No	4.8 (3.6, 6.2)	14.1 (12.3, 16.1)*	1.9 (1.4, 2.4)*	36.9 (35.0, 38.9)*
**Household Language**				
English	4.1 (3.7, 4.5)†	11.4 (10.8, 12.1)†	1.3 (1.2, 1.5)†	32.7 (32.2, 33.2)†
Spanish	4.8 (3.6, 6.4)	15.1 (13.0, 17.5)*	2.8 (1.8, 4.1)*	39.7 (35.3, 44.2)*
Other	3.6 (2.0, 6.4)	13.7 (10.9, 17.0)	2.6 (1.8, 3.9)*	34.6 (31.9, 37.4)
**Chronic Conditions and Disabilities**				
None	4.1 (3.5, 4.7)†	10.3 (9.5, 11.2)†	1.4 (1.2, 1.5)†	29.4 (28.8, 30.0)†
One	4.2 (3.4, 5.1)	12.5 (10.9, 14.3)*	1.5 (1.2, 1.8)	35.0 (33.9, 36.0)*
Two or more	4.4 (3.6, 5.3)	17.0 (15.6, 18.4)*	1.7 (1.4, 2.1)	45.9 (44.7, 47.1)*
**Special Health Care Needs (CSHCN)**				
None	4.1 (3.6, 4.6)†	10.9 (10.2, 11.7)†	1.4 (1.3, 1.6)†	30.7 (30.2, 31.3)†
Any	4.5 (3.7, 5.3)	16.4 (15.0, 17.9)*	1.6 (1.3, 2.0)	44.9 (43.7, 46.0)*

Notes: Survey-weighted predicted probabilities and 95% confidence intervals based on unadjusted and adjusted logistic regression models. **Other race-ethnicity includes American Indian, Alaska Native, Asian Indian, Chinese, Filipino, Guamanian, Japanese, Korean, Native Hawaiian, Samoan, Vietnamese, other Pacific Islander, other Asian, some other race, and multi-racial.†Reference group. *Difference compared to reference group is statistically significant (p<0.05).

© 2023 Daw JR et al. *JAMA Health Forum.*

eTable 4. Inconsistent and Inadequate Insurance for Publicly Insured Children by State, 2016-2021

STATE	INCONSISTENT INSURANCE, ANNUAL WEIGHTED N	INCONSISTENT INSURANCE, WEIGHTED % (95% CI)	INADEQUATE INSURANCE, ANNUAL WEIGHTED N	INADEQUATE INSURANCE, WEIGHTED % (95% CI)
**ALABAMA**	5881	1.4 (0.9, 2.3)	49390	11.9 (9.5, 14.9)
**ALASKA**	2075	4.5 (3.0, 6.8)	5165	11.2 (8.5, 14.7)
**ARIZONA**	28773	5.5 (3.7, 8.1)	64096	12.3 (9.7, 15.5)
**ARKANSAS**	17034	5.9 (4.2, 8.1)	27649	9.5 (7.7, 11.7)
**CALIFORNIA**	102895	3.6 (2.2, 5.8)	375721	13.0 (10.3, 16.2)
**COLORADO**	17031	5.0 (3.4, 7.2)	53876	15.8 (12.8, 19.4)
**CONNECTICUT**	3497	1.8 (0.8, 3.9)	22328	11.7 (8.7, 15.5)
**DC**	927	2.3 (1.2, 4.3)	3929	9.7 (6.9, 13.4)
**DELAWARE**	2025	3.7 (2.4, 5.7)	6936	12.7 (9.9, 16.3)
**FLORIDA**	77468	5.1 (3.4, 7.5)	265710	17.4 (14.7, 20.6)
**GEORGIA**	62957	7.7 (5.7, 10.4)	108610	13.3 (10.7, 16.3)
**HAWAII**	895	1.2 (0.7, 2.1)	7440	10.3 (7.7, 13.6)
**IDAHO**	2317	1.7 (0.9, 3.3)	15770	11.6 (8.8, 15.1)
**ILLINOIS**	32589	3.9 (2.5, 6.3)	163003	19.7 (16.0, 24.0)
**INDIANA**	21269	4.7 (3.0, 7.3)	55818	12.3 (9.5, 15.8)
**IOWA**	5926	3.1 (1.5, 6.2)	20354	10.6 (8.0, 14.0)
**KANSAS**	8283	4.5 (2.7, 7.5)	17405	9.6 (7.1, 12.8)
**KENTUCKY**	7803	2.2 (1.4, 3.4)	30968	8.8 (6.5, 11.8)
**LOUISIANA**	9485	2.2 (1.3, 3.5)	39144	8.9 (7.0, 11.2)
**MAINE**	2740	4.3 (2.6, 7.0)	4862	7.6 (5.6, 10.2)
**MARYLAND**	13471	4.0 (2.6, 6.2)	37858	11.2 (8.3, 15.1)
**MASSACHUSETTS**	8186	2.9 (1.6, 5.0)	31418	11.0 (7.9, 15.0)
**MICHIGAN**	30751	5.1 (3.4, 7.7)	63513	10.6 (8.2, 13.7)
**MINNESOTA**	15475	5.6 (3.4, 9.0)	28777	10.3 (7.5, 14.0)
**MISSISSIPPI**	8931	2.9 (1.9, 4.3)	29975	9.7 (7.9, 11.8)
**MISSOURI**	13664	3.7 (2.1, 6.2)	40208	10.7 (8.3, 13.8)
**MONTANA**	2338	2.9 (1.8, 4.6)	10939	13.6 (10.8, 16.9)
**NEBRASKA**	3559	3.6 (2.3, 5.7)	10291	10.4 (7.4, 14.4)
**NEVADA**	7987	4.6 (2.9, 7.2)	26023	15.0 (11.8, 18.9)
**NEW HAMPSHIRE**	2781	5.0 (3.2, 7.8)	5840	10.5 (7.8, 13.8)
**NEW JERSEY**	22399	4.9 (3.3, 7.2)	62713	13.8 (10.6, 17.7)
**NEW MEXICO**	5589	2.6 (1.6, 4.0)	21347	9.8 (7.9, 12.2)
**NEW YORK**	45928	3.5 (2.2, 5.6)	123514	9.5 (7.4, 12.1)
**NORTH CAROLINA**	30070	3.7 (2.4, 5.8)	88273	11.0 (8.4, 14.2)
**NORTH DAKOTA**	1201	5.5 (3.0, 9.8)	3959	18.2 (13.0, 24.8)
**OHIO**	22096	3.1 (1.7, 5.5)	63225	8.9 (6.4, 12.1)
**OKLAHOMA**	10080	2.9 (1.9, 4.3)	32584	9.4 (7.4, 11.9)
**OREGON**	15050	5.8 (3.8, 8.8)	30043	11.6 (9.1, 14.5)
**PENNSYLVANIA**	17333	2.3 (1.4, 3.8)	59686	8.1 (5.9, 10.9)

© 2023 Daw JR et al. *JAMA Health Forum.*

**RHODE ISLAND**	1741	3.1 (1.7, 5.4)	4472	8.0 (5.7, 11.0)
**SOUTH CAROLINA**	9797	2.5 (1.6, 3.9)	37129	9.5 (7.3, 12.2)
**SOUTH DAKOTA**	2504	5.5 (3.4, 8.7)	5000	11.0 (8.0, 14.9)
**TENNESSEE**	14380	2.9 (1.8, 4.7)	39539	7.9 (6.1, 10.3)
**TEXAS**	147242	6.8 (4.7, 9.6)	297970	13.7 (10.7, 17.2)
**UTAH**	5758	5.4 (2.9, 9.8)	19658	18.3 (13.6, 24.1)
**VERMONT**	260	0.6 (0.3, 1.2)	3340	8.0 (6.2, 10.1)
**VIRGINIA**	15068	3.7 (2.0, 6.8)	45881	11.2 (8.1, 15.2)
**WASHINGTON**	9292	2.1 (1.1, 3.7)	66749	14.8 (11.5, 18.8)
**WEST VIRGINIA**	3914	2.9 (1.8, 4.7)	14951	11.0 (8.6, 14.0)
**WISCONSIN**	11002	3.9 (2.4, 6.1)	32496	11.4 (8.6, 14.9)
**WYOMING**	1320	4.9 (3.1, 7.7)	3312	12.2 (9.3, 15.9)

© 2023 Daw JR et al. *JAMA Health Forum.*

eFigure 2. Inconsistent Insurance for Publicly Insured Children by State, 2016-2021

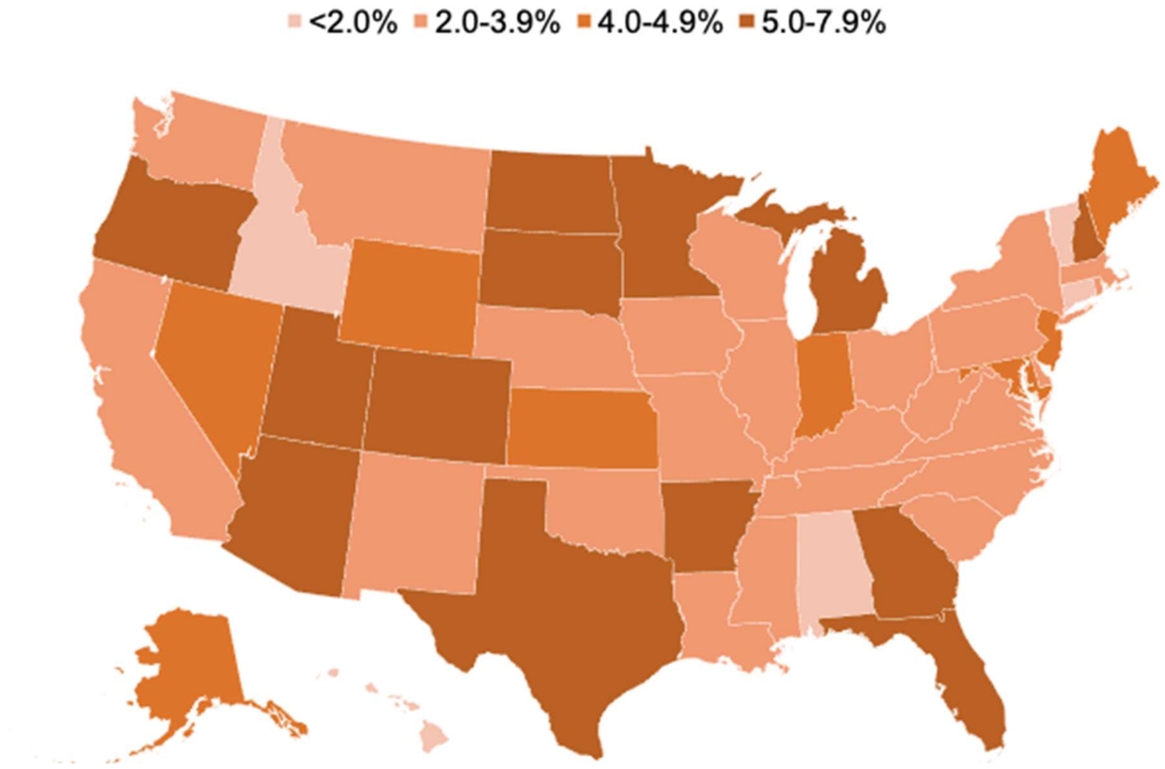

eFigure 3. Inadequate Insurance for Publicly Insured Children by State, 2016-2021

© 2023 Daw JR et al. *JAMA Health Forum.*

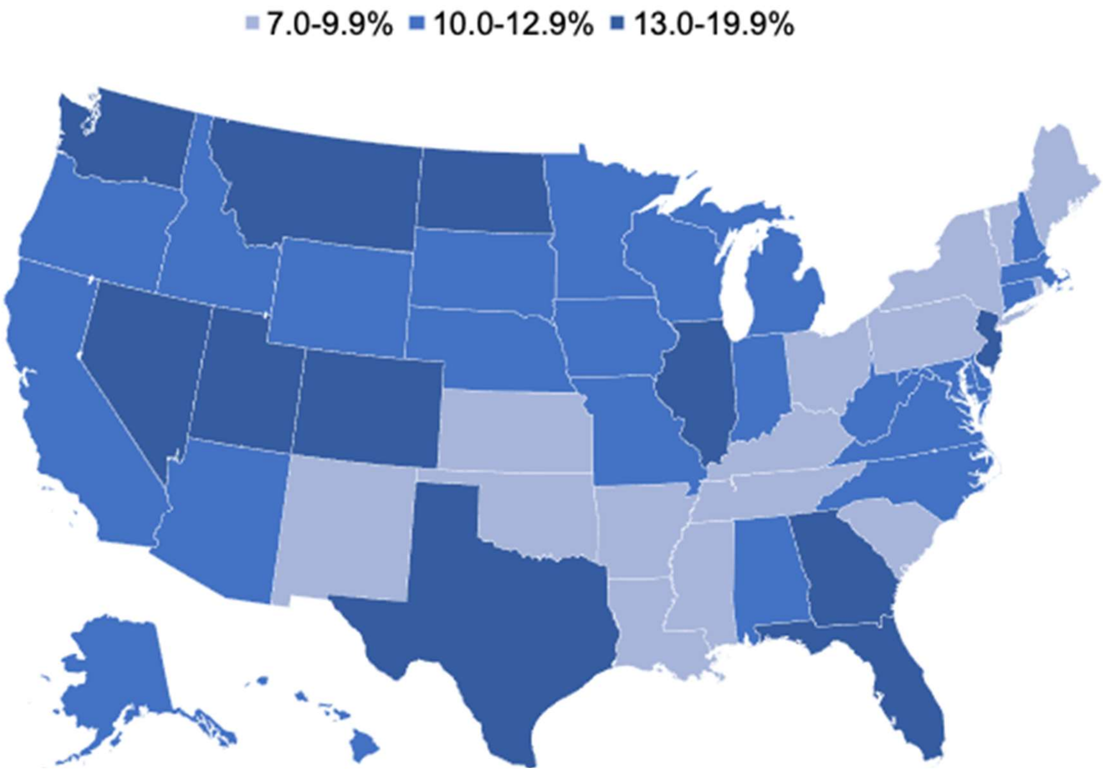

© 2023 Daw JR et al. *JAMA Health Forum.*
