# Supplementary material for: Consistency and Adequacy of Public and Commercial Health Insurance for US Children, 2016 to 2021
Source: JAMA Health Forum. 2023 Nov 22;4(11):e234179. doi: 10.1001/jamahealthforum.2023.4179 (PMC10665966; doi:10.1001/jamahealthforum.2023.4179)
Supplement: Supplement 2. — Data Sharing Statement [file jamahealthforum-e234179-s002.pdf]

Data Sharing Statement

Data
Data available: Yes
Data types: Deidentified participant data, Data dictionary
How to access data: Data and data dictionaries are available from the U.S. Census Bureau: https://www.census.gov/programs-surveys/nsch.html
When available: With publication

Supporting Documents
Document types: None

Additional Information
Who can access the data: All data used in this study is publicly available and can be downloaded on the U.S. Census Bureau website.
Types of analyses: Any purpose
Mechanisms of data availability: All data used in this study is publicly available and can be downloaded on the U.S. Census Bureau website.
